# Mammary sarcoidosis: A rare case report

**DOI:** 10.1016/j.amsu.2022.103892

**Published:** 2022-05-31

**Authors:** Meriem Rhazari, Abdelbassir Ramdani, Sara Gartini, Siham Bouali, Mohammed Aharmim, Afaf Thouil, Hatim Kouismi, Jamal Eddine Bourkadi

**Affiliations:** a Pneumology Department, Mohammed VI University Hospital, Oujda, Morocco; b Mohammed First University Oujda, Faculty of Medicine and Pharmacy Oujda, Oujda, Morocco; c Surgical Oncology Department, Mohammed VI University Hospital, Regional Oncology Center, Oujda, Morocco; d Pneumology Department, Moulay Youssef Hospital – Ibn Sina University Hospital, Rabat, Morocco

**Keywords:** Sarcoidosis, Breast, Granuloma

## Abstract

**Introduction:**

Sarcoidosis is an inflammatory, systemic, idiopathic disease characterized by multisystem involvement, of which mediastinal and pulmonary involvement is the most frequent. Mammary sarcoidosis is exceptional.

**Case presentation:**

We report the case of a 50-year-old, diagnosed with mediastinal and mammary sarcoidosis. Therapeutic abstention with clinical and radiological surveillance was recommended. The evolution was marked by a clear improvement (clinical and radiological).

**Discussion:**

Mammary sarcoidosis is a rare anatomical and clinical entity which poses a problem of differential diagnosis with other granulomatous diseases and especially with breast carcinoma. The coexistence of systemic manifestations should lead to the discussion of sarcoidosis.

**Conclusion:**

Mammary sarcoidosis involvement is rare and is manifested by a mass with a smooth or spiculated border, requiring the exclusion of malignancy.

## Highlights

• Mammary sarcoidosis is a rare clinical entity.
• The final diagnosis is based on a combination of clinical, biological, radiological, and pathological findings.
• The main differential diagnosis of mammary sarcoidosis is tuberculosis and breast cancer.
• Corticosteroid therapy is the reference treatment for breast sarcoidosis.

## 1 Introduction

Sarcoidosis is an immune-mediated disease of unknown etiology, characterized by the presence of noncaseating granulomas, mainly affecting the lungs (over 90%) [1,2].

Breast localization is extremely rare and represents about 1% of cases [2]. The true incidence and prevalence of sarcoidosis worldwide are difficult to determine, as many patients are asymptomatic. It usually occurs among patients with known systemic diseases [3]. However, breast involvement may be the initial site of the disease, manifested by a palpable mass sometimes discovered incidentally on mammography, which must be differentiated from a malignant mass [1]. It constitutes a diagnostic challenge especially with tuberculosis and breast cancer. This case was reported according to the SCARE guideline [4].

## 2 Case report

A 50-year-old diabetic female patient, who has been on oral hypoglycemics for seven years. She had never been treated for tuberculosis and had no recent history of a tuberculosis contact. She reported the appearance of bilateral cervical lymphadenopathy two months before her admission, without any other associated respiratory or extra-respiratory signs, all evolving in a context of fever, night sweats, and preservation of her general condition. The physical examination revealed a patient in a good general condition (PS 1) with multiple lymphadenopathies of different sizes, painless and without any inflammatory signs, located in the cervical, axillary, epitrochlear, and inguinal regions. The rest of the physical examination was unremarkable.

**Fig. 1:**
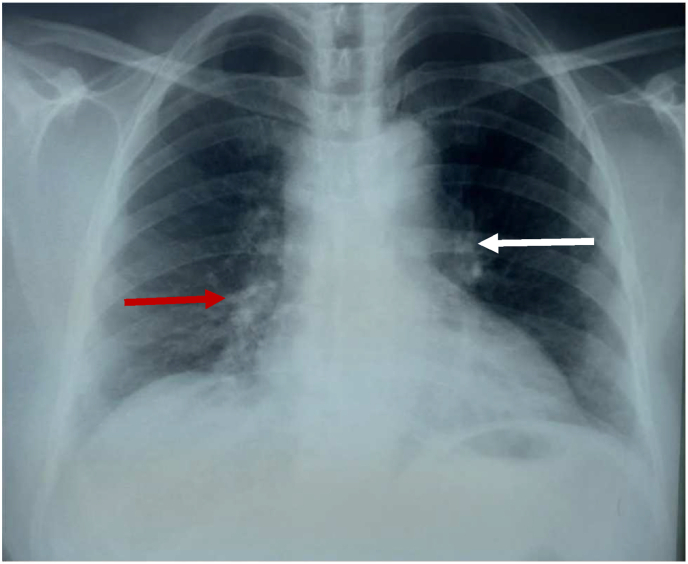
Chest X-ray: Left hilar opacity (white arrow) and a right paracardiac opacity (red arrow). (For interpretation of the references to colour in this figure legend, the reader is referred to the Web version of this article.)

The chest X-ray showed a left hilar opacity and a right paracardiac opacity (Fig. 1). The complete blood count was normal. The C-reactive protein was slightly elevated at 40 mg/l, blood ionogram, kidney function test, liver function test, and the viral serologies including HIV were normal. The search for acid-fast bacilli (AFB) in sputum was negative, and the tuberculin skin test (TST) was positive at 15 mm. The biopsy of the cervical adenopathy showed the presence of an epithelioid and giant cellular granuloma without caseous necrosis. Unfortunately, the culture of the biopsy specimen was not performed.

The patient was put on anti-tuberculosis as tuberculosis was highly suspected due to the epidemiological context, the clinical symptomatology and the strong positivity of the tuberculin test. On the fifteenth day, she developed a generalized pruritic erythematous rash that required the interruption of the treatment. A thoracic CT scan was performed, which revealed mediastinal hilar and axillary adenopathy without parenchymal involvement and a right breast lump (Fig. 2a, Fig. 2b).

**Fig. 2a:**
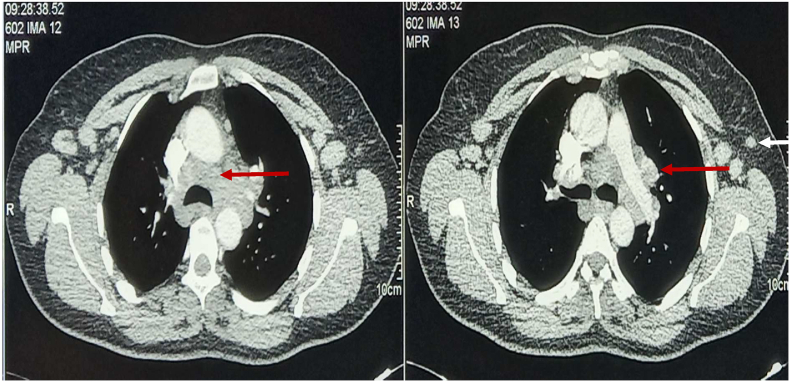
A thoracic CT scan showing non-compressive mediastinal and hilar adenopathy (red arrows) with axillary adenopathy (white arrow).

**Fig. 2b:**
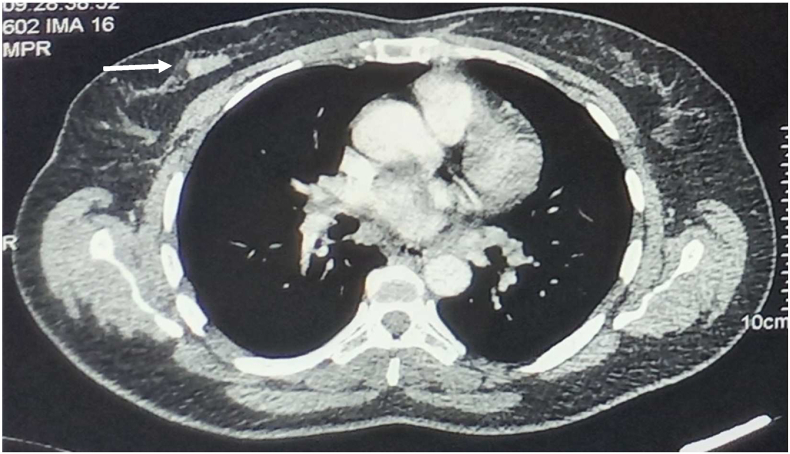
A thoracic CT scan showing a right breast lump (white arrow).

Bronchoscopy showed thickening of the left upper lobar spur, bronchoalveolar lavage was predominantly lymphocytic (42%), staged bronchial biopsies were negative as well as the search for AFB in the bronchial aspiration fluid.

Bilateral mammography and mammary ultrasound showed a right and left breast lump which is classified respectively BI-RADS V and BI-RADS IV (Fig. 3a, Fig. 3b). The breast biopsy was in favor of a granulomatous inflammatory process without caseous necrosis.

**Fig. 3a:**
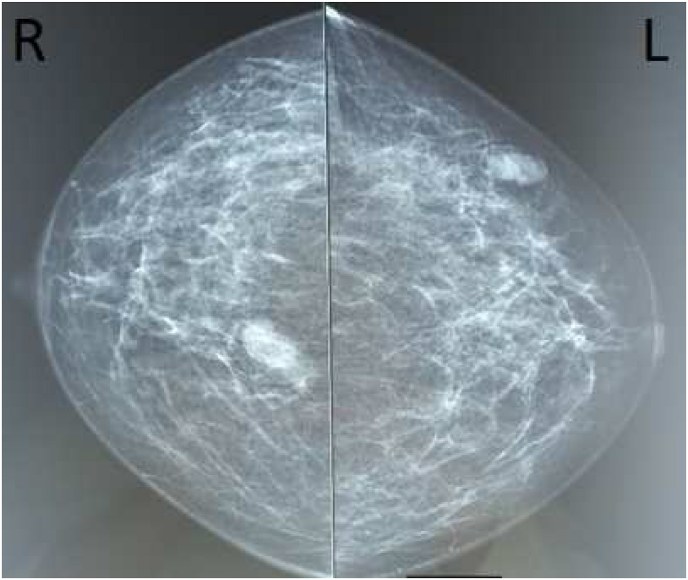
A bilateral mammography: A right and left breast lump which are classified respectively ACR V and ACR IV.

**Figure 3b:**
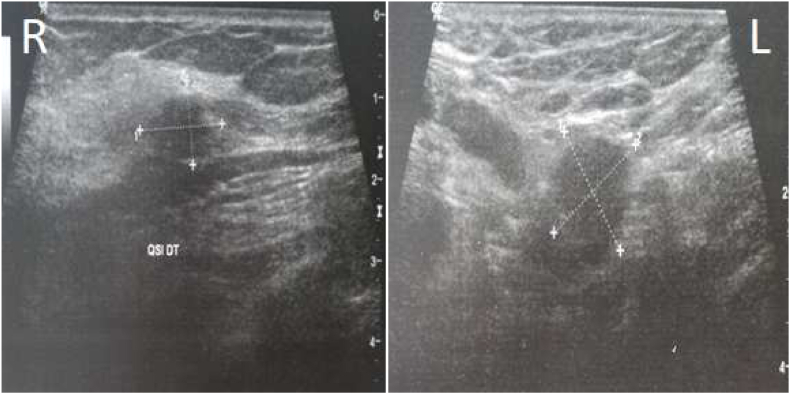
Mammary ultrasound: A right and left breast lump which are classified respectively BI-RADS V and BI-RADS IV.

The phosphocalcic balance, the electrocardiogram, and the ophthalmic exam were without abnormalities. The biopsy of the accessory salivary glands showed the presence of a giant cellular epithelioid granuloma without caseous necrosis compatible with sarcoidosis. The pulmonary function test showed a correct total lung capacity (TLC). Beta microglobulinemia, serum protein electrophoresis, and abdominal ultrasound were without abnormalities.

The diagnosis of sarcoidosis was based on clinical, radiological, biological, and histological criteria (presence of granuloma without caseous necrosis in 3 different organs: lymph node, salivary glands, and mammary glands).

**Fig. 4:**
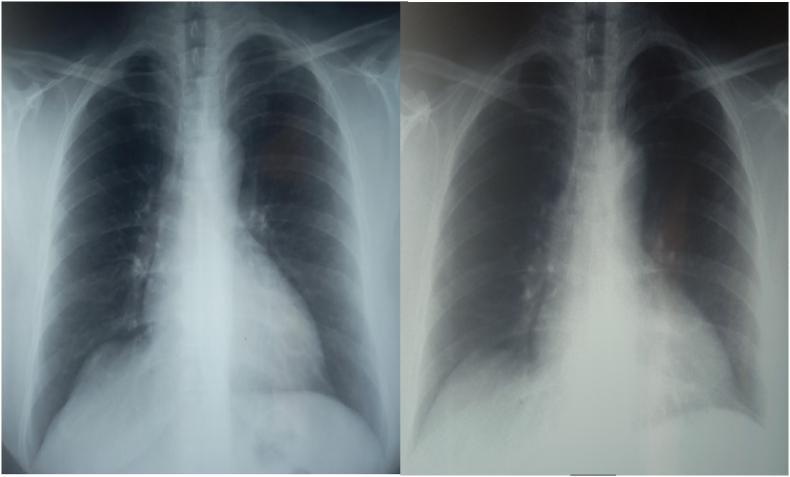
Chest X-ray: improvement of radiological lesions over two years.

Therapeutic abstention was recommended with clinical and radiological surveillance. The evolution was marked over two years by the disappearance of arthralgias, hilar adenopathies (Fig. 4) and peripheral adenopathies.

## 3 Discussion

Sarcoidosis is a granulomatous pathology with multi-systemic involvement that is pathologically manifested by the formation of non-caseating epithelioid granulomas [2]. It mainly affects people in the third and fourth decades of life, but can also appear among children and the elderly [5]. The true incidence and prevalence of sarcoidosis worldwide are difficult to determine, as many patients are asymptomatic. A higher incidence of the disease in northern countries (about 60 per 100,000) than in southern European countries [5]. The disease is more common in women than men, nonsmokers than smokers, and in some races, such as African Americans, with familiar forms in less than 10% of cases [5,6].In France, mortality is estimated at 3.6 per million habitats, usually related to pulmonary fibrosis and respiratory failure [7].

The etiology of sarcoidosis is still poorly understood; numerous investigations into the existence of an infectious agent that may induce the disease have been conducted in recent years. The frequent presence of mycobacterial DNA has been reported in patients with sarcoidosis. Other infectious agents have also been incriminated, such as herpes or Epstein-Barr virus (EBV), but also other bacteria such as Borrelia burgdorferi. Other theories have also emerged, including exposure to environmental (insecticides, molds) [8].

Sarcoidosis is a systemic inflammatory disorder that is characterized by an immune response involving T-helper cells in which CD4 lymphocytes and activated macrophages accumulate in the affected organs, leading to granuloma formation. An undiscovered antigen, processed by activated macrophages, induces an immune response modulated by T cells and macrophages that release a broad spectrum of mediators, including cytokines, chemokines, and oxygen radicals involved in the etiopathogenesis [9].

Breast involvement in sarcoidosis is rare and represents about 1% of cases [2]. It may manifest as a single or multiple mass, varying in size from 0.5 cm to 8 cm, unilateral or bilateral, mobile and painless, with edges that may be smooth or irregular, requiring the exclusion of malignancy. Sometimes discovered incidentally on screening mammography [1,10]. In our case, the discovery of the mass was incidental in the thoracic CT scan.

Mammography may show well-limited heterogeneous opacities or single or multiple spiculated opacities simulating a malignant mass, small round well-defined masses have also been described, reflecting intramammary lymph node involvement, and ultrasound may show the mass as well or poorly limited hypoechoic opacities [11,12]. Breast MRI may point to the neoplastic origin of the mass by showing inhomogeneous signal intensity, with irregular contours and fast enhancement with early fading [1].

The diagnostic criteria for sarcoidosis include a suggestive clinical and radiological background, demonstration of epithelioid and giant cellular granuloma without caseous necrosis, and elimination of other known causes of granuloma (Table 1). Bronchoalveolar lavage has a diagnostic orientation value but does not affirm it, by an increase in the percentage of lymphocytes (between 30 and 50%) [13].

**Table 1:** Main differential diagnoses of sarcoidosis [13].

Cause	Pathology	Main diagnosis elements
Infections	Tuberculosis, histoplasmosis, leprosy, Whipple's disease and others	Epidemiological context, contagion, presentation, microbiology
Environmental agents	Berylliosis Hypersensitivity pneumonitis	Anamnesis and presentation #, beryllium hypersensitivity (TTL in blood or BAL), genetic susceptibility (HLA-DPß1 glutamate 69) Anamnesis, presentation, precipitins
Medication	INF γ and β, anti-TNF therapy, BCG intra-vesical therapy, immune restoration under anti-retroviral therapy, CTLA-4, anti-PD-1 or PDL1 inhibitors …	Anamnesis
Immunodeficiency	common variable immunodeficiency Chronic Granulomatous disease	Presentation, repeated infections, hypogammaglobulinemia, Family history, repeated infections, phagocytosis defect, functional tests, genetic abnormalities of CYBB, NCF1, NCF2, CYBA or NCFA
Genetic disease	Blau's syndrome	Pediatric onset, family history, presentation, NOD genetic anomaly
Malignant diseases	Epithelial cancers and seminomas Hodgkin's disease and lymphoid granulomatosis	Anatomopathology Anatomopathology, molecular biology
Others	Granulomatosis with polyangiitis Crohn's disease Primary biliary cirrhosis	Presentation, ANCA, anatomopathology Presentation, endoscopies Presentation, immune assessment test

Tuberculin anergy is present in 75% of cases; positive TST does not exclude sarcoidosis, as in our patient's case where a positive TST was found. The diagnostic value of serum angiotensin-converting enzyme is low [14,15]. The presence of hypercalciuria, more rarely hypercalcemia, or peripheral lymphopenia may help to orient the diagnosis. The presence of polyclonal hypergammaglobulinemia is frequent; it is particularly useful in distinguishing sarcoidosis from systemic granulomatosis associated with common variable immunodeficiency [16].

Histological investigations should be performed in a hierarchical manner [13]. The sensitivity of staged bronchial biopsies for the detection of tuberculoid granulomas is about 60%–80% [17]. Transbronchial lung biopsies obtained by bronchoscopy have a sensitivity up to 90% [18]. Peripheral lymph nodes, skin, and even conjunctival biopsies are often preferred. The sensitivity of accessory salivary gland biopsies is about 20%–40% in patients with predominantly thoracic involvement [13]. Video-surgical lung biopsy is rarely required [16]. In the case of breast involvement in sarcoidosis, a biopsy is necessary both to exclude breast cancer and for the diagnosis of sarcoidosis [2]. Samples from other sites may be discussed depending on the clinical manifestations (skin, muscle, liver) [13].

The differential diagnosis is mainly with other granulomatosis (Table 1) and in particular with tuberculosis, especially in an endemic country, and in front of a breast mass the differential diagnosis is mainly breast cancer [1,13].

Tuberculosis and sarcoidosis are both granulomatosis diseases that have similar clinical and radiological aspects that are sometimes very difficult to differentiate between the two pathologies, many cases of tuberculosis are initially misdiagnosed as sarcoidosis and vice versa [19]. As in the case of our patient, the diagnosis of tuberculosis is retained clinically (positive TST). In addition, there are several patients diagnosed with tuberculosis and sarcoidosis concomitantly [20].

In this context, the diagnosis of tuberculosis is established by a positive culture from body fluids or tissues, but may not be possible in some organs because of difficulties in non-invasive access or because of the inability to obtain results. In this case, the positivity of interferon-gamma release assays (IGRA) and tuberculin intradermal reaction at a threshold of 13 mm remains strongly associated with tuberculosis infection with a sensitivity of 54% and specificity of 90% [19,21].

The granuloma without caseous necrosis, the main but non-specific lesion, is composed of macrophages, epithelioid and giant cells in the center, in close contact with CD4^+^ T lymphocytes [3]. Around these granulomas, a lymphocytic corona (CD8^+^ T cells, regulatory T cells, Th17 and B cells) is organized [13]. However, necrosis has been reported up to 30% in sarcoidosis. Similarly, non-caseating granulomas can be found in tuberculosis, so there are no specific features to differentiate the two diseases with certainty, except for a positive culture of mycobacterium tuberculosis [19].

In our patient, non-necrotizing granulomas were found in three different sites (lymph node, salivary gland, and breast). Corticosteroid therapy is the reference treatment for breast sarcoidosis, allowing a satisfactory clinical and radiological improvement [1]. Immunosuppressive drugs are indicated in the other localization of sarcoidosis that can be functional or life-threatening [22]. Our patient was put under clinical and radiological surveillance. The evolution was marked by a clear clinical and radiological stability.

## 4 Conclusion

Breast sarcoidosis is rare and appears clinically as a palpable mass or incidentally discovered on screening mammography. It has a differential diagnosis problem with breast cancer and tuberculosis, so histological examination should be mandatory to establish an early and conclusive diagnosis of breast sarcoidosis.

## Ethics approval

No ethical approval necessary.

## Source of funding

The author(s) received no financial support for the research, authorship and/or publication of this article.

## Author contribution

**Meriem Rhazari, Abdelbassir Ramdani:** Writing, review and editing of the manuscript.**Sara Gartini, Siham Bouali, Mohammed Aharmim, Afaf Thouil, Hatim Kouismi:** Contributed for diagnose and treatment of the patient.**Jamal Eddine Bourkadi: S**upervised the writing of manuscript.

## Trial registry number

Our paper is a case report; no registration was done for it.

## Guarantor

Meriem Rhazari, Abdelbassir Ramdani.

## Consent

Written informed consent was obtained from the patient for publication of this case report and accompanying images. A copy of the written consent is available for review by the Editor-in-Chief of this journal on request.

## Provenance and peer review

Not commissioned, externally peer reviewed.

## Data availability

Data will be made available on request.

## Declaration of competing interest

The authors declared no potential conflicts of interests with respect to research, authorship and/or publication of the article.
